# Ruptured liver cavernous hemangioma – rare cause of hemoperitoneum

**Published:** 2015

**Authors:** OC Goidescu, T Patrascu

**Affiliations:** * Surgery Department, Municipal Hospital, Ramnicu Sarat, Romania; ** ”I. Juvara” Clinical Hospital, “Dr. I. Cantacuzino” Hospital, Bucharest, Romania

**Keywords:** cavernous hemangioma liver hemoperitoneum, liver cirrhosis

## Abstract

We present the case of a 70-year-old patient admitted with strong abdominal pains and operated in our service for hemorrhagic acute abdomen. Intraoperative hepatic cavernous hemangioma was found with capsular rupture and hemoperitoneum. Due to liver cirrhosis, and no proper technical equipment we chose to perform simple hemostasis. Postoperative evolution was favorable.

## Introduction

Cavernous hemangioma is the most common benign tumor of the liver, incidence in different studies ranging from 0.7% to 7%. The discovery is most often incidental on ultrasound examinations for other complaints or at necropsy. The symptoms are polymorphic and can often be attributed to other diseases. More than 4 cm sized giant hemangioma is considered but it may reach sizes of 10-12cm. The therapeutic attitude varies from simple clinical supervision to medication and surgery generally reserved for symptomatic cases [**5**]. The complication most feared is rupture; the spontaneous rupture is extremely rare, 21 cases being reported in literature.

## Case Report

A 70-year-old patient was admitted in our service for intense abdominal pain, sudden onset approximately 2 hours before admission, apparently due to non-traumatic etiology. From the patient's personal history: L4-L5 disc herniation surgery, he also described increased consumption of NSAIDs and ethanol. On admission, the patient was pale, BP 90/55 mmHg, heart rate 100bpm, tachypnea, mild ethanol halitosis, abdominal muscular defense. We tried to perform an ultrasound examination, which found liquid in the peritoneal cavity but we could not finish the ultrasound examination, the patient becoming unstable and he being taken to the operative room. The biological samples on admission showed anemia, Hb 8.7 g/ dl, Ht 28.8%, mild leukocytosis 11600/ mmc, increased levels of liver enzymes ALT 103 U/ L and AST 280 U/ L, and the presence of hepatitis B virus. Later, changes of electrophoresis α2 increased to 13.9% and γ to 27.7% were found. Emergency surgery was performed; intraoperatively the presence of approximately 1200ml blood was noticed in the peritoneal cavity, liver with micronodules appearance next round ligament insertion formation of cavernous hemangioma with rupture of the capsule and active bleeding. Given the urgency, the hemodynamic unstable patient, the macroscopic appearance of the liver and lack of preoperative investigations and technical equipment, hemostatis with sponges and drainage was chosen. Intra and postoperatively, the patient was transfused with 3 units MER and 3 units FFP. Postoperatively, the evolution was favorable, but the patient presented ascites in the first postoperative days, up to 1500ml then slowed down to extinction, the patient being discharged 22 days after surgery. At discharge, liver enzymes were AST 40 U/ L, ALT 90 U/ l and Hb 10mg/ dl. The patient refused to be admitted in a specialized center in liver surgery, came to control one time after discharge.

**Fig. 1:**
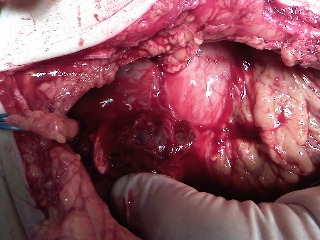
Intraoperative aspect

## Discussion

Hemangiomas are one of the most common liver tumors, second after liver metastases. There are extensive discussions in literature regarding the therapeutic attitude. The symptoms in these conditions are highly variable from pain, anemia, inflammatory manifestations, bleeding, blood disorders and, in adults, rarely Kasebach-Merrit syndrome [**1**,**3**] consisting coagulopathy due to coagulation and fibrinolysis inside hemangioma.

Ultrasound and CT investigations remain elective, MRI being useful for tumors <2cm. Also, there are useful hepatic angiography and biopsy, the last one with high risk of hemorrhage. These patients need extensive investigations to rule out any conditions that can cause the symptoms described by the patients. Treatment is surgical and nonsurgical. Ultrasound follow up and various methods of treatment including interferon α, steroids, arterial embolization, radiation therapy, are used for asymptomatic hemangiomas [**4**]. Preoperative embolization can be used to lower the blood supply. Surgery is not currently indicated by the size of the hemangiomas but by the complications that may occur: blood disorders, diagnostic uncertainty, hemorrhage, necrosis inside the tumor. Surgical methods are enucleation, liver resection, hepatic artery ligation and liver transplantation [**2**]. In the case described we could not use these methods due to liver disease and technical equipment limitations. We opted for a minimal solution to remove the patient from the emergency situation.

## Conclusion

We were facing a particular situation of hemorrhagic acute abdomen in a patient with major liver disease due to both toxic-nutritional and viral causes. The etiology of the rupture was still uncertain, the limited degree of cooperation and not very accurate historical data provided could not exclude trauma, even in the absence of traumatic abdominal marks. The minimal solution chosen was beneficial for the patient, long-term prognosis remained limited due to pre-existing liver disease, eating habits and lack of cooperation.

**Disclosure:** none
